# Education Intervention on Chronotherapy for Final-Year Pharmacy Students

**DOI:** 10.3390/pharmacy3040269

**Published:** 2015-11-04

**Authors:** Gagandeep Kaur, Maya Saba, Craig L. Phillips, Keith Wong, Bandana Saini

**Affiliations:** 1 Faculty of Pharmacy, The University of Sydney, Camperdown, NSW 2006, Australia; E-Mails: maya.saba@sydney.edu.au (M.S.); bandana.saini@sydney.edu.au (B.S.); 2 Woolcock Institute of Medical Research, Glebe, NSW 2037, Australia; E-Mails: craig.phillips@sydney.edu.au (C.P.); keith.wong@sydney.edu.au (K.W.)

**Keywords:** chronotherapy, circadian rhythms, intervention, pharmacy students, work shop, education

## Abstract

Chronotherapy involves altering the timing of medication administration in coordination with the body’s circadian rhythms to improve the overall control of disease and to minimise treatment side effects. Training on chronotherapy requires students to map different topics learnt in earlier years of their professional degree and apply these concepts clinically. This requires strategic educational design. Therefore, the aim of the study was to develop, implement and evaluate an educational intervention focusing on the application of chronotherapy for final-year undergraduate pharmacy students. An educational intervention utilizing multiple learning strategies for enhancing chronotherapy related awareness was designed and implemented in the final year undergraduate pharmacy cohort at the University of Sydney Australia (2013). A custom-designed questionnaire measuring awareness about (13 items scored 0 or 1), and attitudes (12 items scored on a Likert scale of 1–5) towards chronotherapy was administered pre and post intervention to evaluate its impact. The pre-intervention mean total awareness and attitude scores were 6.5 ± 2.0 (score range 0–13) and 47.4 ± 6.9 (score range 12–60) respectively. The mean total post-intervention scores were significantly higher for total awareness (10.1 ± 1.9) and attitude (54.0 ± 6.0). Carefully designed educational interventions utilising pedagogic principles for pharmacy students can improve awareness of and enhance positive attitudes toward pharmacists’ roles in optimizing drug therapy using chronotherapy.

## 1. Introduction

Clinical knowledge about biological rhythms, the importance of the sleep-wake routine, and the effect of circadian timing on medicine administration is significant for the management of particular disease conditions and for potentiating the therapeutic effects and/or reducing the adverse effects of certain medications. Broadly referred to as chronotherapy, the administration of medications at optimal circadian times represents a multifaceted concept that exploits diverse mechanisms and principles such as formulation science, pharmacology, physiology, and pathophysiology [1,2]. Though not applicable in all cases, chronotherapy may be of significance in disease conditions that exhibit circadian variability such as asthma, gastroesophageal reflux, cardiovascular diseases, cancer, metabolic diseases, rheumatoid arthritis and sleep disorders [3,4,5,6]. Further, evidence from the various clinical effectiveness and drug disposition studies demonstrates improved outcomes when the administration of certain medications is timed in synchrony with circadian patterns [4,7,8]. A prime example is that of the statins, where short-acting statins are more effective when administered at night time, the circadian point when cholesterol synthesis is at its peak and HMG-CoA reductase enzyme exhibits maximal activity [9].

With recent advances in sleep and circadian medicine, awareness of chronotherapeutic principles has been growing, and clinicians have started to incorporate this research evidence into clinical practice [10]. As primary health professionals, community pharmacists can undoubtedly play a significant role in this area. This is particularly pertinent since community pharmacists often represent the last point of contact with patients prior to medication administration. Similarly, in hospital settings, pharmacists utilize their broad therapeutic expertise to optimize patient medication regimens [11]. Given that pharmacists are among the most accessible healthcare professionals who can provide medication and health-related advice without the need for appointment or consulting fees, patients are highly likely to seek pharmacists’ advice on various health-related matters, including queries about the most appropriate time for administering a particular medication [12].

Although it may be one of several niche concepts in the pharmacists’ armament, chronotherapy represents a novel way of optimizing the patient experience of medications and delivering individualized care. In Australia, the Pharmacy Guild, a national body representing community pharmacies, highlighted the importance of timing of drug administration to support patient adherence to their prescribed treatment over time [13,14]. The Accreditation Standards for Pharmacy Programs in Australia and New Zealand require pharmacy degree programs to establish a curriculum that equips students with the knowledge and skills to ensure the safe and effective use of medicines in consumers. [15]. Therefore, it is important that students, as future pharmacists are equipped with up-to-date knowledge about recent research in various clinical areas related to medication use including, for example, chronotherapy. As future pharmacists, they need to be well-positioned to educate their patients about the optimal timing of medication administration, chronotherapeutic technologies, and circadian and pathophysiological advantages of chronotherapy.

Although the fields of chronobiology and sleep and circadian rhythms have been around since the 1950s [16], until recently in clinical pharmacy circles it was believed that evidence for this concept was in equipoise. This concept was thus not usually covered in depth in clinical pharmacy courses [17], except at a very practical level [18]. Most pharmacists and pharmacy students may be aware, for example, that medications that interfere with sleep should be taken earlier in the daytime, *i.e.*, a single-dose diuretic might be better taken in the morning rather than at night. However, a deeper understanding of the concepts underpinning chronotherapy and exposure to newly emerging data is not included in most undergraduate pharmacy programs. This may be linked to the novelty of the sleep and circadian medicine field itself, which is only now being introduced in nursing and medical education in a meaningful manner [19,20].

Given recent evidence, supporting chronotherapy, education and training to improve pharmacy professionals’ understanding of this concept is essential. Since chronotherapy represents an advanced clinical concept requiring learners to cross-link and apply their previous basic understanding of several underlying concepts in pharmacology, physiology and therapeutics, educational experiences constructed to diffuse awareness about chronotherapy need to be carefully planned with attention to pedagogical principles. Further, given the novelty of the concept, it may be likely that for many drugs, specific chronotherapeutic information would not be available, hence future graduates would need the skills to apply training in chronotherapy to such drugs, so that drug administration times are circadianly optimised for enhanced safety or efficacy. The final year curriculum would be the best place to situate such training, as learners at this stage would have the required pre-requisite learning, and with imminent entry to the profession, they would be keen to learn knowledge application skills. Therefore, the aim of the study was to develop, implement, and evaluate an education intervention focusing on principles of chronotherapy for final year undergraduate pharmacy students.

## 2. Experimental Section

### 2.1. Design of Education Intervention

The main purpose of the educational intervention was to provide students with evidence-based knowledge of principles on which chronotherapy is based and to enhance their awareness of the application of these principles in pharmacy practice. The study was approved by the Human Research Ethics Committee at the University of Sydney (HREC number 2012/2885).

The research design used to evaluate this experiment was a pre-post evaluation, where the evaluation instrument comprised a custom devised questionnaire. The questionnaire was used to assess participants’ knowledge of circadian rhythms and chronotherapy and their attitudes and willingness to apply principles of chronotherapy throughout their professional life. The construction of the questionnaire was based on a review of the literature [2], a previous survey (1996 Gallup survey of medical chronobiology and chronotherapeutics) [21] and practical considerations in the clinical application of chronotherapy. It comprised three sections as highlighted in Table 1. Experienced practicing pharmacists, pharmacy academics, sleep and circadian specialists reviewed the questionnaire for face and content validity. The questionnaire was pilot tested with five practicing pharmacists. Prior to the questionnaire administration, all final-year pharmacy students (*n* = 216) enrolled in the “Pharmacy Management” (PHAR 4814) course during 1 Semester 2013 (Faculty of Pharmacy, University of Sydney, Australia) were offered a verbal invitation to participate in the study and requested to complete this questionnaire. For an implementation plan timed for the 2nd Semester 2013 (July–October), the questionnaire administration in Semester 1 allowed researchers to map knowledge deficits and construct an intervention that addressed existing needs. The same questionnaire was then administered after the intervention was delivered in the 2nd Semester 2013 as well.

**Table 1:** The content of the utilized questionnaire.

Section	Questionnaire Section	Description
1	General Demographics and Information Section	9 items that included statements with Yes/No answers and short questions covering demographic and general data about the students’ work experience and information on the chronotherapy.
2	Awareness Section	13 items that included brief statements or clinical case studies with multiple-choice response options. The possible score for each item was either 0 or 1. The total possible score range for the section was 0–13.
3	Attitude Section	12 items that included statements with multiple options covering attitudes and willingness to use principles of chronotherapy. Each item was measured using the Likert scale of “Strongly Agree”, “Somewhat Agree”, “Neither Agree Nor Disagree”, “Somewhat Disagree”, and “Strongly Disagree”. The possible score range for each item was 1 to 5. The total possible score range for the section was 12–60.

When developing a method to provide the education, it was hypothesized that the participation in a multi-component interactive educational intervention would increase students’ knowledge about and attitudes towards the concept of circadian rhythms and principles of chronotherapy Compared to traditional directive teaching, interactive educational experiences enable students to become more active, independent and self-directed learners. Ultimately, this enhances their learning process by facilitating appropriate application of their knowledge in new contexts [22]. Thus, the educational intervention was designed based on the principles of active learning [23], and the learning outcomes were couched in accordance with Bloom’s taxonomy of cognitive constructs [24]. The learning objectives, teaching activities and assessment tasks were aligned carefully (Table 2).

The educational intervention protocols and educational artifacts were developed by the first author in consultation with academics and clinicians who have extensive expertise in pharmacy education research, and sleep/circadian medicine. The educational activity was embedded in an existing practice based course on Professional Pharmacy (PHAR 4821). This course, offered to final year students, focuses on knowledge consolidation, therapeutics, application of knowledge, continuing professional education practices, and disease-state management. It consists of lectures, hands-on workshop sessions, online learning, and problem-based tutorial classes [18]. A detailed description of the intervention is provided below.

**Table 2:** Learning objectives and alignment with instruction and assessment plans.

Learning Objectives	Instructional Strategies	Type of Learning (*Bloom’s taxonomy*)	Assessment Strategy
Define the key terms used in the field of circadian rhythms and chronotherapy.	Knowledge transfer through the delivery of a didactic lecture. Provision of references to background articles in the workshop	Factual knowledge/Remembering	Performance in post-workshop questionnaire and exam questions
Understand the significance of circadian rhythms in various disease conditions	Delivery of the lecture. Screening of a BBC documentary and a few research articles as background references in the workshop	Comprehension	Discussion towards the end of the lecture and workshop. Performance in post-workshop questionnaire and exam questions
Apply the scientific rationale behind the timing of administration of medicine	Delivery of a lecture. Screening of a BBC documentary and a few research articles as background references in the workshop. Discussion of case studies and role-play during the workshop	Comprehension	Assessing the quality of case discussion, *i.e.*, how learners provided convincing support or evidence for the basis of their proposed case solution throughout their presentation. Performance in post-workshop questionnaire and exam questions
Demonstrate an understanding of different aspects of chronotherapy in terms of pharmacokinetics, dynamics, formulation and effectiveness	Delivery of a lecture. Provision of in-depth information on various aspects of chronotherapy. Discussion of case studies during the workshop	Application, Analysis, and Synthesis	Assessing the quality of case discussion, *i.e.,* how learners provided convincing support or evidence for the basis of their proposed case solution throughout their presentation. Performance in post-workshop questionnaire and exam questions
Compare the limitations and benefits of chronotherapy applications in clinical practice	Delivery of a lecture. Provision of in-depth information on different aspects of chronotherapy. Discussion of case studies, poster-making and role-play during the workshop	Analysis	Observation and analysis of case studies, poster and role-play. Peer critique, followed by group discussion.
Justify the role of pharmacists in implementing principles of chronotherapy and optimize drug therapy.	Discussion of case studies, role-play and poster making	Analysis, Evaluation, and Skill Acquisition	Observation and analysis of case studies, poster and role-play. Peer critique, followed by group discussion

#### 2.1.1. “Medications Around the Clock”—Lecture

A 1-h lecture entitled “Medications around the clock” was prepared following a thorough review of the literature. The main purpose of the lecture was to provide the students with an overview of circadian rhythms, key definitions, and concepts used in chronobiology and chronotherapy. A meta-cognitive approach was applied in the construction and presentation of the lecture. The lecture, thus, covered fundamental concepts ranging from linking chronotherapy terms and explaining mechanisms of how chronotherapy may work to highlighting the latter relationally with case examples in a laddered approach, *i.e.*, proceeding from simple to more complex matters [25,26]. In other words, during the lecture, the lecturer provided “knowledge” or “facts” based on chronotherapy—and then linked these in (1) a backward integration to knowledge/concepts learnt in previous years and (2) a forward integration to how the knowledge could be applied in a clinical case patient presenting to a pharmacy. This sequence was used to direct cognitive processes in learners in their learning effort. The lecture was delivered by an experienced clinical pharmacist (last author) to 216 final-year pharmacy students in the fourth week of studies during the 2nd Semester 2013. An open discussion addressing questions and stimulating feedback was encouraged at the end of the lecture. The lecture was recorded and posted online along with the corresponding presentation slides (Microsoft PowerPoint^®^ format) for future study reference.

#### 2.1.2. “Medications Around the Clock—Chronotherapy”—Workshop

A 3-h workshop entitled “Medications around the clock—Chronotherapy” was carefully designed to reinforce and “scaffold” the knowledge acquired from the lecture [27]. It consisted of multiple collaborative and group learning activities including a video screening, case studies, poster making, and role plays. Table 3 describes the content of the workshop. The workshop was delivered by the first and last authors during the final week (week 13) of studies in the 2nd Semester 2013. The workshop took place in an allocated room over a period of one week and was administered to 192 final-year pharmacy students in “Professional Pharmacy” course. The subject coordinator for PHAR 4821 had divided the students into six groups of 30–34 students each, and each group was allocated to a single workshop session in the last week of the semester. On the day of any particular workshop, the instructors gave an introduction, outlining the objectives and content of the workshop. Following this, screening of a short, publicly available, British Broadcasting Corporation (BBC) documentary video was carried out [28]. This was intended to stimulate a relaxed learning environment and allow audio-visual engagement with the topic. Following the screening, students were allocated to one of the four groups, referred to as “stations”; with each station comprising 7–9 students. Information packages containing group activity details, research articles on the subject, and relevant medical references were provided for each station. Each station received a different set of activities covering 1 of 4 themes: (1) Concepts in chronotherapy; (2) Chronotherapy of pain; (3) Chronotherapy of hypertension; and (4) Chronotherapeutic drug delivery systems. The workshop room was also equipped with computers/other resources to facilitate the students’ research.

**Table 3:** The content of the workshop.

Activity (Time Allotted for the Activities) ^a^	Objective	Description
Video screening of a BBC documentary (30 min)	Introducing the audio-visual support for scaffolding clinical learning	BBC documentary: “Horizon - The Secret life of your biological clock” was screened. The documentary explained how the human biological clock works and how it affects and controls routine life activities. The video helped grasp students’ interest and engagement in the topic through the visualization of real-life scenarios.
Clinical case studies and poster making (100 min: 40 min for solving the cases and 60 min for class discussion)	Clinical concept learning-collaborative learning, critical thinking, and generating a new idea in a team environment.	Clinical case studies related to circadian rhythms and chronotherapy principles that could be encountered in future practice were provided to each group. During the first 40-min, each group was asked to read, discuss, and analyze their case. The key clinical query was to determine an appropriate time for administering the medication in question. Students in each station were then asked to design a single poster addressing awareness of chronotherapy with their patients. Then 60 min were allocated for class discussion of the case studies. The case studies were presented using multimedia screen and read out by corresponding students from each station. Discussions covered issues such as counseling patients about the right time of administration, explaining to patients about the circadian variability in symptoms of disease conditions in lay language, and differences between conventional drug delivery systems and chronotherapeutic drug delivery systems. Each station displayed their poster on a multimedia screen, and an instructor-led the discussion. This activity was engaging and collated various ideas whilst providing learners with a creative outlet. The instructor guided the discussion by asking questions and answering queries from the students. All groups were encouraged to take part in the debate and provide input.
Role Play (10 min)	Collaborative learning-Practicing acquired knowledge.	A group of students role-played the “patient”, the “pharmacist” and observer from clinical case studies. In the role play, the “pharmacist” counsels the “patient” regarding the “right time” of administration of medicines and promoting chronotherapy application in pharmacy.
Debriefing (10 min)	Solidifying learning concepts, synthesizing new knowledge	The instructor initiated the discussion by summarizing key points covered in the lecture and throughout the workshop focusing on variability in circadian rhythms and timing of medication administration.

Note: (**^a^**) 10 min were allotted at the beginning for the introduction and a brief description of the workshop; 20 min at the end of the workshop was reserved for the assessment.

Each station had a set of clinical case studies allocated to their topic; these were designed to integrate scientific and collaborative learning by improving student’s clinical knowledge and practice skills related to the timing of drug administration. Table 4 summarizes the content of clinical cases. Students at each station also presented their “solutions” to their set cases to the entire class. The question-answer session that followed the presentations stimulated collaborative learning with different groups learning from each other (as each station had different cases). Some clinical cases were role played out; a structured approach to role-playing was utilized by rotating students through the roles of the pharmacist, patient, and observer. Each role helped the students acquire new communication skills via adopting different perspectives. In one station, an activity requiring students to develop a poster (to explain chronotherapy in lay terms) provided an opportunity to generate new ideas in a creative manner and to practice and refine presentation skills. Every element of the workshop was designed to encourage a highly interactive learning environment. The instructors provided assistance by answering questions, facilitating task completion, and guiding the flow of the workshop activities. The overall instructional design followed an upward trajectory, *i.e.*, learning was commenced at lower levels of Bloom’s taxonomy of cognitive constructs (didactic lecture) and proceeded to higher cognitive levels [29].

**Table 4:** Examples of clinical case studies in the workshop.

Case Studies	Clinical Expertise Tested
*Chronotherapy of pain*: A 40-year-old male presents with epigastric burning pain. His physician prescribed omeprazole 40 mg for 14 days and advised him to change his diet.	Counseling for administration (right time of administration), lifestyle modification, contraindications, precautions, and storage
*Chronotherapy of pain*: A 49-year-old male had been waking up during the night-time with heartburn and reflux. The physician advised Antacid and zantac 300 mg.	Counseling for administration (right time of administration), lifestyle modification, contraindications, precautions, and storage.
*Chronotherapy of pain*: A 33-year-old female gradually developed painful wrists and early morning stiffness. The physician prescribed her ketoprofen 200 mg.	Counseling for administration (right time of administration), lifestyle modification, contraindications, precautions, and storage.
*Chronotherapy of hypertension*: A 62-year-old male has a past medical history of hypertension. His 24h ambulatory blood pressure indicates that nighttime blood pressure is about 25% lower than his daytime blood pressure.	Addressing patients’ concerns, therapy selection/counseling, lifestyle modifications.
*Chronotherapy of hypertension*: A 52-year-old female has a history which includes type-2 diabetes, hypertension, and obstructive sleep apnea. Her 24 ambulatory blood pressure monitoring indicates that her blood pressure declines less than 10% during the night compared with daytime.	Addressing patients’ concerns, therapy selection/counseling, lifestyle modifications.
*Chronotherapeutic drug delivery systems*: A 50-year-old female who presents to the pharmacy with a new prescription for Covera-HSR (verapamil).	Counseling for administration (right time of administration, how to take the medication), lifestyle modification, contraindications, precautions, and storage.
*Concepts in chronotherapy*: After an acute asthma attack, a 32-year-old woman is prescribed 40 mg of prednisone daily.	Counseling for administration (right time of administration), lifestyle modification, contraindications, precautions, and storage.

## 3. Results and Discussion

### 3.1. Evaluation and Assessment of Education Intervention

Pedagogical procedures should be carefully planned and continuously examined [30]. During the course of workshop, students’ performance was continuously examined by the instructors by means of observing their in-class progress which included completion of assigned tasks, their participation in group discussions, their queries, enthusiasm and attentiveness to the presentation.

To evaluate the impact of the educational activity on students’ knowledge about and attitudes toward chronotherapy and its application, the pre-intervention questionnaire was administered after the implementation of the education activities, at the completion of the workshops. During the administration of both the pre and post intervention questionnaires, the students were requested to complete their questionnaires within 10–20 min without discussing or seeking help from each other nor checking any relevant resources for responses. The questionnaire was administrated by the first author.

Data from the questionnaires was collected and analyzed using the Statistical Package for Social Sciences (version 22.0, IBM, Armonk, NY, USA). A total of 212 students completed the pre-workshop questionnaire (response rate 98%) and 192 students completed the post-workshop questionnaire (response rate 100%). Complete paired data sets were available for 157 students as 35 students did not provide their allocated identification code correctly, which resulted in utilizing a sample size of 157 students for statistical analysis purposes. Out of 157 students, 98.7% were under the age of 30, and 62.4% were females. Seventy-nine percent of the students were employed at pharmacies, and only 12.7% had previous knowledge about chronotherapy. Mean total awareness (knowledge) and attitude scores were calculated for the 13-item awareness section and the 12-item attitude sections items of the questionnaire. The awareness questions were scored either 1 (correctly answered) or 0 (incorrectly answered). The attitude questions were scored on 5 point Likert scale from “strongly disagree” to “strongly agree”. Item with strongly agree scored 5 at one end and strongly disagree scored a 1 at the other end of the Likert scale. Pre and post-intervention scores were compared using paired *t*-tests. The pre-intervention mean total awareness and attitude scores were 6.5 ± 2.0 (score range 0–13) and 47.4 ± 6.9 (score range 12–60) respectively. The post-intervention total awareness score was 10.1 ± 1.9, and the attitude score was 54.0 ± 6.0. Post-intervention scores were significantly higher than pre-intervention scores with a score difference of 3.6 (95% CI, 3.2–3.9) and 6.6 (95% CI, 5.6–7.7) respectively.

From an educational perspective, pre and post intervention item difficulty and discrimination indices of the awareness section of the questionnaire were calculated [31,32]. The mean item difficulty, which corresponds to the proportion of students who answered an item correctly, was calculated to be 0.50 for pre-intervention and 0.78 for post-intervention. Item discrimination indices were used to review the ability of the awareness items of the questionnaire to discriminate between respondents who scored at either end of the scoring spectrum. It was computed from equal-sized high and low scoring groups to measure the ability of a particular item to differentiate between students with high scores and those with low scores. Item discrimination index was calculated to be 0.37 for pre-intervention and 0.33 for post-intervention.

To further evaluate the content of the provided educational activity, students’ feedback was sought anonymously using a feedback form at the end of the workshop. The form comprised open-ended questions and 5-point Likert scale responses evaluating four key criteria of the workshop: (1) quality of teaching; (2) content of supporting material; (3) learning objectives; and (4) relevance of the workshop to future pharmacy practice. Mean scores were calculated for the Likert scale responses while the open-ended questions were thematically analyzed.

A total of 160 students completed the provided feedback form. Table 5 summarizes the results of the Likert scale questions. The majority of students (95%) highlighted the significance of chronotherapy-related education and advocated its retention in the curriculum for final-year pharmacy students. Most of the students reported that the introduction of a video screening was unique and made the workshop activities more engaging.

“Great introduction video to introduce the mood of the workshop.”

“The tutors were very helpful, the video was interesting, the workshop was fun and we could see the relevance of it for our careers as pharmacists.”

Many students also reported that the workshop was comprehensive and relevant to future practice:

“I found the workshop very informative. It helped settle down all the pieces of what I have learned in the last four years. It provided me with the practical advice that can be used in real practice. I totally enjoyed the workshop.”

“It really opened my eyes more on the reasoning of time in relation to dosing regimen.”

**Table 5:** Result of the feedback evaluation.

Feedback	Mean (SD) *
The workshop meets the stated learning objective.	2.1 (1.4)
The supporting materials used in the workshop (video, reference materials, PowerPoint template) were useful, and they enhance my learning experience.	2.1 (1.4)
The instructors used appropriate teaching techniques to enhance my learning.	1.8 (1.4)
Overall, the workshop was relevant and useful for my future practice.	2.1 (1.5)

Note: * The score was based on a Likert scale from 1–5, where 1 is strongly agree, and 5 is strongly disagree.

As a follow-up to the provided educational intervention, the subject of chronotherapy was then included at the end of semester examinations of final-year pharmacy students. Four questions (3 multiple choice and 1 short-answer) on the subject were included in “Professional Pharmacy Final Exam” (PHAR 4821) that took place at the end of the 2nd Semester 2013.

The students’ overall performance at the end of the semester examination was favorable. Most students handled the multiple choice question well and proposed optimal therapeutic plans for the short descriptive question. The mean item difficulty for the three multiple choice exam questions in the final exam was 0.73. This result indicated considerable improvement in knowledge about chronotherapy. Item discrimination for the three multiple choice questions using the point bi-serial correlation was 0.37, 0.39 and 0.31; this is within the acceptable range of 0.3–0.6 [31].

### 3.2. Discussion

This research study reports on the design of an educational intervention on chronotherapy for final-year undergraduate pharmacy students. The aim of the study was to assess students’ awareness and understanding of circadian rhythms and principles of chronotherapy and provide them with up-to-date education on this advanced clinical concept. Given that not much is known about effective methods for teaching students about this advanced concept, the design of an educational intervention with careful attention to pedagogic principles was warranted. This was achieved by balancing a spectrum of educational components ranging from didactic lectures to interactive case discussions within a scaffolding structure of contextual support materials. A significant improvement in pharmacy students’ awareness of principles of chronotherapy and their application was observed following the exposure to the 4-h educational intervention. The improvement was significant in terms of both general knowledge about circadian rhythms and clinical-based information on priniciples of chronotherapy which was evident from improvements in scores of awareness items of questionnaire, class performance in the workshop and scores at end semester examination. The intervention highlighted that complex clinical aspects can be successfully delivered using well-designed educational interventions.

While the pre-intervention attitude score was already high, a further improvement was observed after the implementation of the educational activity. This indicated that pharmacy students were enthusiastic and motivated to learn new skills that could assist them in optimizing pharmacotherapeutic outcomes throughout their professional career. The delivered intervention, thus, facilitated the students’ learning and achieved the desired outcomes. With respect to knowledge gains, an increase in the mean item difficulty and a decrease in the discrimination index values post-intervention were also observed. This indicates that, after the education, the proportion of awareness questions answered correctly was higher, and the gap between high-scoring and low-scoring students was reduced. These results may be attributed to the pedagogic design of the intervention. This could also highlight the sensitivity of the utilized questionnaire to the established changes in knowledge. Additionally, students’ feedback on teaching standards, course organization, and the interactive nature of the workshop were overwhelmingly positive and highlighted the careful attention paid to the construction of the intervention. This resulted in the inclusion of a chronotherapy module in the curriculum of undergraduate pharmacy students at the University of Sydney (e.g., 2014 onwards).

From an instructional perspective, several studies have demonstrated that educational interventions with teaching methods fostering students’ participation and engagement could enhance knowledge and positive attitudes [22,32]. Various learning techniques were, thus, employed during the design of the educational intervention. For instance, the use of professionally-designed, audio-visual tools to scaffold student learning was an effective strategy, integral to the flow and design of the intervention. Most students reported that the use of a video documentary was interesting and highly engaging, and they recommended its future use in other academic courses. Videos have, in fact, been used in continuing education training for pharmacists and as educational tools for pharmacy students [33]. Studies have shown that videotapes are effective in teaching clinical methods to medical students [34,35]. For example, videos have been used to introduce particularly difficult subjects, such as psychological adaptation to terminal illnesses and to illustrate the progressive changes that may occur in chronic disease conditions within a limited course schedule [36]. Another learning strategy utilized in our intervention was the employment of small-group activities and discussions. Small group formats have been particularly useful in medical education [37]. Studies have shown that small group discussions allowed students to apply their knowledge in solving problem-based case studies and to develop clinical and professional skills and reflective discussions [38]. Moreover, the role-play technique that we utilized has been regularly used in several studies to build communication skills [39] and was found out to be useful in improving the confidence of pharmacy students and pharmacists when counseling patients about common diseases and medications [22]. The literature also indicates that artwork and drawing have been efficient in building stronger communication skills [40]. The results of a study, in which students collaborated in pairs to research a public health topic related to pharmacy practice, demonstrated that a poster creation activity was effective in increasing knowledge and awareness of public health issues [41]. Similarly, in our intervention, the poster-making activity helped students to improve their verbal presentation abilities, drug information skills, and the ability to collaborate with other team members. All in all, the use of these combined learning strategies stimulated the students’ critical thinking, clinical decision-making, and communication and counseling skills, particularly about the timing of drug administration and circadian rhythms.

It should be noted this study had a number of limitations. The questionnaire utilized to assess students’ knowledge and attitudes was developed by the authors for the purpose of this study and was not validated or previously psychometrically-tested. Since the workshop was conducted in the last week of semester, the post-intervention questionnaire had to be administered on the same day of the workshop; hence, the recency of learning may have elicited the positive responses obtained. A possible limitation also includes test-retest bias as the same survey instrument was used to assess students’ awareness and attitudes both before and after the education. To add, a follow-up study to examine the degree of knowledge retention was not conducted. In future research, it would be wise to do further content validation through administrating the questionnaire to sleep and circadian experts and to follow up with the students after they become registered pharmacists, possibly through the use of questionnaires or simulated patient scenarios.

## 4. Conclusions

An educational intervention on chronotherapy for pharmacy students was successful in improving awareness of circadian rhythms and principles of chronotherapy and enhancing attitudes toward the application of these principles in pharmacy practice. After the conclusion of this study, a two-hour chronotherapy module has been adopted into the curriculum of final-year pharmacy students at the University of Sydney. While interactive teaching techniques have been found to be useful in training pharmacy students on chronotherapy, similar strategies should be employed in the design of an adapted chronotherapy-based educational intervention or training program for pharmacy practitioners, possibly as part of their continuing professional education program. Other healthcare professionals, such as general practitioners, physicians, and practice nurses may also benefit from a similar intervention.
